# Unraveling the Predictive Power: Placenta Growth Factor and Pregnancy-Associated Plasma Protein A in Pre-eclampsia

**DOI:** 10.7759/cureus.52752

**Published:** 2024-01-22

**Authors:** Aditi Singh Thakur, Surekha Tayade, Drashti Patel, Aishwarya Gupta, Nitish Batra

**Affiliations:** 1 Obstetrics and Gynecology, Jawaharlal Nehru Medical College, Wardha, IND; 2 Medicine, Jawaharlal Nehru Medical College, Wardha, IND

**Keywords:** therapeutic interventions, angiogenic imbalance, biomarkers, pregnancy-associated plasma protein a (papp-a), placenta growth factor (plgf), pre-eclampsia

## Abstract

This review provides a comprehensive exploration of the roles of placenta growth factor (PlGF) and pregnancy-associated plasma protein A (PAPP-A) in the context of pre-eclampsia, a pregnancy-related hypertensive disorder with significant implications for maternal and fetal health. The background elucidates the clinical significance of pre-eclampsia, highlighting its prevalence and impact. The review delves into the biological importance of PlGF and PAPP-A, emphasizing their critical roles in normal placental development and their dysregulation in pre-eclampsia. Notably, altered levels of these biomarkers emerge as potential diagnostic indicators, offering insights into the pathophysiology of the disorder. The exploration of pathophysiological mechanisms, including angiogenic imbalance and placental dysfunction, provides a nuanced understanding of pre-eclampsia's molecular landscape. The therapeutic implications of targeting PlGF and PAPP-A open avenues for future research, aiming at effective intervention strategies. The conclusion summarizes key findings, outlines implications for future research, and underscores the crucial role of PlGF and PAPP-A in understanding and managing pre-eclampsia, with the ultimate goal of improving outcomes for both mothers and infants.

## Introduction and background

Pre-eclampsia is a pregnancy-related hypertensive disorder characterized by the onset of high blood pressure and significant organ dysfunction, typically occurring after 20 weeks of gestation. It remains a leading cause of maternal and fetal morbidity and mortality worldwide, affecting approximately 5-8% of pregnancies. The pathophysiology of pre-eclampsia involves abnormal placental development, impaired maternal vascular response, and systemic inflammation [1]. Despite decades of research, the precise etiology of pre-eclampsia remains elusive, making early detection, management, and prevention challenging. Understanding the molecular and biochemical factors associated with this condition is crucial for improving both maternal and fetal outcomes [2].

Placenta growth factor (PlGF) and pregnancy-associated plasma protein A (PAPP-A) are two key biomolecules intricately involved in the regulation of normal placental development and function. PlGF, a member of the vascular endothelial growth factor (VEGF) family, plays a pivotal role in angiogenesis and vascular endothelial function during pregnancy. PAPP-A, on the other hand, is a metalloproteinase that regulates insulin-like growth factor bioavailability, contributing to placental growth and fetal development [3]. The dysregulation of PlGF and PAPP-A has been implicated in the pathogenesis of pre-eclampsia. Altered levels of these biomarkers have been observed in pre-eclamptic pregnancies, suggesting their potential utility as diagnostic and prognostic indicators for this disorder [4].

The purpose of this comprehensive review is to delve into the intricate interplay between PlGF and PAPP-A in the context of pre-eclampsia. By synthesizing existing literature and recent advancements in the field, this review aims to provide a thorough understanding of the roles these biomolecules play in normal pregnancy and how their dysregulation contributes to the pathophysiology of pre-eclampsia. Additionally, this review will explore the diagnostic and prognostic potential of PlGF and PAPP-A, their molecular mechanisms in pre-eclampsia, and the therapeutic implications that arise from targeting these biomarkers. Through a critical analysis of current research, the review seeks to contribute to the ongoing efforts to improve pre-eclampsia prediction, management, and ultimately, maternal and fetal outcomes.

## Review

Pre-eclampsia: definition and epidemiology

Definition and Diagnostic Criteria

Pre-eclampsia is a progressive multisystem disorder characterized by the sudden onset of hypertension and proteinuria, or the sudden onset of hypertension coupled with significant end-organ dysfunction, with or without proteinuria. Typically, this condition manifests after 20 weeks of gestation or postpartum [5]. The diagnostic criteria for pre-eclampsia encompass a systolic blood pressure equal to or exceeding 140 mmHg or a diastolic blood pressure equal to or exceeding 90 mmHg, measured on two separate occasions with at least a four-hour interval. Additionally, proteinuria of 300 mg or more in a 24-hour urine specimen or a urine protein-to-creatinine ratio of 0.3 or more is considered diagnostic [6-8]. It is crucial to note that proteinuria is no longer an obligatory criterion for pre-eclampsia diagnosis. Other indications of end-organ dysfunction, such as thrombocytopenia, impaired liver function, renal insufficiency, pulmonary edema, or the emergence of new cerebral or visual disturbances, can also be utilized for diagnosis [6,7]. It is imperative to include HELLP syndrome, a severe form of pre-eclampsia involving hemolysis, elevated liver enzymes, and low platelet count, among the critical manifestations. The incidence of pre-eclampsia varies widely among different populations, but it is estimated to impact 2-8% of pregnancies globally [7].

Incidence and Risk Factors

Pre-eclampsia, a condition affecting 2% to 10% of pregnancies globally, exhibits varied incidence rates across developing and developed countries, with reports indicating approximately 1.8-16.7% in the former and 0.4% in the latter [9]. Numerous factors contribute to the risk of developing pre-eclampsia, highlighting the complex nature of this disorder. Firstly, a significant determinant is a woman's medical history, as those who experienced pre-eclampsia in a previous pregnancy face a substantially higher sevenfold risk of recurrence [10]. Additionally, chronic conditions such as high blood pressure, kidney disease, and preexisting diabetes significantly elevate the likelihood of developing pre-eclampsia [5,10].

Furthermore, the association between obesity and an increased susceptibility to pre-eclampsia underscores the role of lifestyle factors in maternal health [10]. Age is another crucial factor, with women older than 40 facing a higher risk of developing pre-eclampsia [10]. Pregnancy-related factors such as first pregnancies, multiple gestations, and assisted reproductive technologies are also linked to an elevated risk [10]. Ethnicity and family history further contribute to the complexity of pre-eclampsia risk, as African American ethnicity and a family history of the condition are associated with a higher likelihood of its occurrence [10]. Moreover, specific health conditions, including migraines, rheumatoid arthritis, lupus, and sickle cell disease, are identified as additional risk factors [10]. However, it is important to acknowledge the inadequacies in documenting the prevalence of pre-eclampsia, especially in certain regions where data are scarce. This emphasizes the critical need for further research and increased awareness, particularly in developing countries [11]. Understanding these risk factors is essential for effective preventive measures, timely diagnosis, and improved management of pre-eclampsia, ultimately enhancing maternal and fetal outcomes.

Impact on Maternal and Fetal Health

Pre-eclampsia exerts a profound impact on the health of both the mother and the fetus. Without proper intervention, it can result in severe complications for women, including acute renal failure, liver failure, and a heightened risk of stroke. Furthermore, if left unchecked, pre-eclampsia may progress to eclampsia, a condition characterized by seizures [12]. The repercussions extend beyond the immediate postpartum period, with long-term effects on both maternal and child health. Mothers who have experienced pre-eclampsia face an increased susceptibility to hypertension and chronic kidney disease [13]. Moreover, pre-eclampsia poses a substantial threat to fetal well-being, significantly elevating the risks of perinatal mortality and morbidity, encompassing stillbirths and neonatal deaths [14]. This risk is particularly pronounced in developing countries, where the prevalence of pre-eclampsia is high, contributing to a greater likelihood of obstetric complications and maternal mortality compared to developed nations [11]. Optimizing fetal outcomes in pre-eclampsia cases requires effective neonatal care facilitated by well-equipped neonatal intensive care units and proficient pediatricians [14]. Early diagnosis and vigilant monitoring are pivotal in managing pre-eclampsia during pregnancy, offering opportunities for timely intervention and control [11]. In the realm of predictive and diagnostic tools, placental growth factor and PAPP-A show promise in anticipating the onset of pre-eclampsia. Utilizing these biomarkers holds potential for early diagnosis and effective management of this complex condition [15].

PlGF

Structure and Function

PlGF is a noteworthy protein belonging to the VEGF family with a pivotal role in pathological angiogenesis [16]. This protein, encoded by the PGF gene and discovered in 1991, functions as a glycosylated homodimer with six cysteine residues in each monomer [16-18]. Primarily secreted in the placental trophoblast, PlGF exerts pro-angiogenic effects on the fetoplacental circulation, supporting trophoblast growth [19]. Despite its primary expression in the placenta, PlGF is also found at lower levels in various tissues, including the heart, lung, thyroid, liver, and kidney [19]. The significance of PlGF extends beyond its physiological functions, as it has garnered attention for its potential predictive power in pre-eclampsia. This hypertensive disorder poses serious complications for both the mother and the fetus, making the exploration of biomarkers like PlGF crucial for early detection and intervention [19]. The multifaceted nature of PlGF, with its role in angiogenesis and trophoblast growth, underscores its potential as a valuable diagnostic tool and an avenue for further research in understanding and managing pre-eclampsia.

Regulation of PlGF Expression

The intricate regulation of PlGF expression remains a subject of ongoing investigation, with various mechanisms under exploration. One study has identified the protein kinase A pathway as a mediator in the regulation of PlGF gene expression within placental villi and trophoblast cells [20]. Additionally, another study has proposed the involvement of endoplasmic reticulum stress and epigenetic changes in influencing PlGF expression [19]. PlGF, in exerting its effects, employs diverse mechanisms such as intermolecular transphosphorylation, heterodimer formation with VEGF, and binding to neuropilin receptor-1 [19]. Its heightened expression throughout all gestational stages in the placenta suggests a role in controlling the growth and differentiation of trophoblastic cells [21]. Notably, the presence of a heparin-binding domain implies that PlGF-2 and -4 maintain cell membrane association, while diffusible forms of PlGF likely exert their effects in a paracrine manner [21]. A prominent structural feature of PlGF arises from the six cysteine residues in each monomer, highlighting its distinct molecular architecture [18]. While the full spectrum of regulatory mechanisms governing PlGF expression is yet to be fully elucidated, these insights into its structural and functional attributes contribute to the ongoing understanding of its roles in placental biology and its potential implications in health and disease.

Physiological Roles in Pregnancy

Throughout pregnancy, PlGF assumes a crucial role in placental development, actively supporting the growth of trophoblasts [22]. Predominantly expressed in the placental trophoblast, PlGF is also found in various tissues, albeit at lower levels [22]. The significance of PlGF extends beyond its physiological functions, as it has been the subject of extensive study due to its potential as a predictive marker for pre-eclampsia hypertensive disorder, fraught with severe complications for both the mother and the fetus [22]. In tandem with PlGF, pregnancy triggers a cascade of physiological changes that collectively contribute to the optimal development of the fetus and prepare the mother for labor and delivery. These changes encompass alterations in hormone levels, augmented maternal blood volume, increased cardiac output, and elevated blood flow to the kidneys and uteroplacental unit [23]. These adaptations collectively ensure a supportive environment for the developing fetus and create the necessary physiological conditions for a successful labor and delivery [22-24]. The intricate interplay between PlGF and the broader spectrum of physiological changes underscores the complexity of the maternal-fetal dynamic during pregnancy.

Altered Levels in Pre-eclampsia

Dysregulation of PlGF levels has been identified in women affected by pre-eclampsia, a pregnancy-specific disorder associated with severe complications for both the mother and the fetus [25]. The hallmark of pre-eclampsia lies in insufficient placentation, a consequence of inadequate trophoblastic invasion into the uterine spiral arteries [26]. This deficiency results in placental hypoxia, the release of proinflammatory cytokines, and the secretion of both angiogenic and antiangiogenic factors [26]. An imbalanced ratio between these factors is a characteristic feature of pre-eclampsia and is implicated in the disease's pathogenesis [25]. Two such factors, soluble fms-like tyrosine kinase-1 (sFlt-1) and PlGF, have emerged as subjects of study due to their potential predictive capabilities for pre-eclampsia [27]. Elevated levels of sFlt-1 and reduced levels of PlGF have been identified as precursors that can predict the subsequent development of pre-eclampsia, with these alterations detectable several weeks before the syndrome manifests [26]. Simultaneous assessments of sFlt-1, PlGF, and uterine perfusion in pregnant individuals have shown promise in predicting early-onset pre-eclampsia with a notable 83% accuracy [26]. Recognizing altered PlGF levels in pre-eclampsia emphasizes the importance of understanding the delicate balance between angiogenic and anti-angiogenic factors in disease development. Despite the progress made in predictive markers, further research is essential to deepening our comprehension of PlGF's role in the pathophysiology of pre-eclampsia. Enhanced knowledge in this domain is key to refining existing diagnostic tools and developing more accurate predictive measures for timely intervention and management.

Molecular mechanisms in pre-eclampsia

Angiogenic Imbalance

Pre-eclampsia, an exclusive pregnancy disorder, is characterized by the onset of hypertension and proteinuria after 20 weeks of gestation. A crucial molecular mechanism linked to pre-eclampsia is angiogenic imbalance, which involves alterations in circulating angiogenic factors. This imbalance is notable for an increase in anti-angiogenic proteins like sFlt-1 and soluble endoglin (sEng), alongside a reduction in pro-angiogenic factors such as PlGF [28]. Research suggests that sFlt-1, an endogenously produced anti-angiogenic protein by the placenta, operates by binding to and inhibiting pro-angiogenic factors, particularly VEGF and PlGF. This disturbed balance in angiogenic factors contributes to endothelial and organ damage, leading to clinical manifestations like hypertension and proteinuria in pre-eclampsia [28-30]. Crucially, the angiogenic imbalance is not confined to the clinical onset of pre-eclampsia but precedes symptoms by several weeks. It is proposed that evaluating and rectifying this imbalance could have substantial clinical implications, influencing the prediction, diagnosis, and management of pre-eclampsia [30]. The intricate molecular mechanisms underpinning pre-eclampsia, particularly the characterized angiogenic imbalance in circulating angiogenic factors, play a pivotal role in the disorder's pathogenesis. A nuanced comprehension and assessment of this imbalance may carry significant implications for predicting, diagnosing, and effectively managing pre-eclampsia.

Placental Dysfunction

Placental dysfunction: In the context of pre-eclampsia, a notable factor contributing to the disorder is placental dysfunction. The placenta overproduces sFlt-1 in this condition. This excess sFlt-1 binds to circulating VEGF and PlGF, hindering their interaction with endothelial cell-surface receptors. The consequence of this interference is the onset of endothelial dysfunction, a pivotal element in the development of pre-eclampsia [28]. The compromised endothelial function plays a central role in the vascular complications associated with pre-eclampsia.

Inflammation: Another facet in the multifaceted pathogenesis of pre-eclampsia involves inflammation. Inflammatory cytokines and maternal infections are believed to contribute to the development of pre-eclampsia. The inflammatory response can induce endothelial dysfunction, further intertwining inflammation with the pathogenesis of pre-eclampsia [31]. The intricate interplay between the immune system and endothelial function underscores the complexity of this disorder and its systemic impact on maternal and fetal health.

Angiogenic factors: Essential for developing blood vessels in the placenta, angiogenic factors like VEGF and PlGF play a pivotal role. In pre-eclampsia, evidence suggests reduced maternal spiral artery conversion, leading to abnormal maternal-fetal interactions and placental malperfusion. This, in turn, triggers an increased secretion of sFlt-1 and a reduction in PlGF levels. The altered balance of these angiogenic factors contributes significantly to the pathogenesis of pre-eclampsia [32]. Understanding the intricacies of this disruption sheds light on the vascular and placental complications inherent in the disorder.

Intrauterine growth restriction (IUGR): In the spectrum of pre-eclampsia-related complications, IUGR is a significant concern. IUGR is characterized by fetal growth retardation, and its association with the development of pre-eclampsia is acknowledged. While the precise mechanisms linking IUGR to pre-eclampsia remain incompletely understood, there is a suggestion that an imbalance of sFlt-1 and PlGF may be involved. This underscores the interconnectedness of various factors contributing to the complex landscape of pre-eclampsia [31]. Further research is imperative to unravel the intricacies of this relationship and enhance our understanding of the disorder's etiology.

Maternal infection: Maternal infection has emerged as a potential contributory factor in the development of pre-eclampsia. The inflammatory response triggered by infection can induce endothelial dysfunction, playing a significant role in the pathogenesis of pre-eclampsia. The intricate interplay between infection-induced inflammation and endothelial dysfunction adds a layer of complexity to our understanding of this pregnancy-specific disorder [31]. Comprehensive research is essential to delineate the specific mechanisms through which maternal infection influences the development of pre-eclampsia.

Obesity and metabolic disorders: Linkages between obesity, metabolic disorders, and the onset of pre-eclampsia have been recognized, with conditions such as gestational diabetes mellitus identified as potential risk factors. The exact mechanisms underlying this association are not yet fully elucidated but may involve an imbalance of sFlt-1 and PlGF [31]. Unraveling the intricacies of how obesity and metabolic disorders contribute to pre-eclampsia is crucial for developing targeted interventions and preventive strategies.

Transforming growth factor-beta (TGF-β): TGF-β, known for its anti-inflammatory and vasodilator properties, assumes a significant role in normal physiological processes. However, in pre-eclampsia, the elimination of TGF-β by sEng disrupts its regulatory function, contributing to endothelial dysfunction [32]. The involvement of TGF-β in the molecular mechanisms of pre-eclampsia introduces an additional layer of complexity, highlighting the intricate network of factors contributing to this disorder. Further exploration of the TGF-β pathway in the context of pre-eclampsia is warranted for a more comprehensive understanding.

Inflammatory Responses

Excessive inflammatory response: In pre-eclampsia, women affected by this condition often display an exaggerated inflammatory response stemming from the dysregulation of the endogenous immune system [33]. This hyperactive inflammatory state can precipitate immune imbalances and inflict damage on vascular endothelial cells, creating a conducive environment for the emergence of pregnancy complications associated with pre-eclampsia [33]. The cascade of events triggered by this excessive inflammatory response underscores its pivotal role in the pathogenesis of pre-eclampsia, contributing to the overall systemic impact on maternal and fetal health.

Inflammatory cytokines: Pre-eclampsia is intricately linked to the production of autoantibodies and heightened expression of endothelin-1, both of which contribute to the development of hypertension during pregnancy and the disruption of placental function [34]. These inflammatory cytokines create a pro-inflammatory milieu, further complicating the already intricate dynamics of pre-eclampsia. The altered placental function and elevated blood pressure are indicative of the far-reaching consequences of inflammatory processes in this pregnancy-specific disorder.

Placental ischemia and immune imbalance: The intricate interplay between immune cells, such as inflammatory macrophages, dendritic cells, NK cells, and T cells, assumes significance in early-onset pre-eclampsia. In this scenario, these immune cells disrupt the formation of spiral arteries, leading to placental hypoxia [35]. Late-onset pre-eclampsia presents a different dynamic, where normal spiral artery formation coexists with insufficient perfusion capacity, resulting in placental hypoxia and a reminiscent inflammatory environment akin to early-onset pre-eclampsia [35]. This intricate connection between immune responses and placental perfusion underscores the multifaceted nature of pre-eclampsia's pathophysiology.

Memory of inflammation: In a compelling aspect, inflammatory cells from pre-eclampsia retain a memory postpartum, thereby elevating the risk of experiencing pre-eclampsia in subsequent pregnancies [35]. This phenomenon signifies the persistence of inflammatory alterations beyond the immediate gestational period, emphasizing the long-term implications of inflammatory responses in the recurrence of pre-eclampsia. The intricate mechanisms underpinning the memory of inflammation postpartum remain an area that warrants further exploration.

Oxidative Stress

Oxidative stress plays a pivotal role in the pathophysiology of pre-eclampsia, stemming from defective trophoblast invasion and a reduction in placental perfusion. This results in an imbalance between the generation of reactive oxygen species and the antioxidant defense system [36,37]. Such an imbalance induces dysfunction in endothelial cells, thereby contributing to the onset of pre-eclampsia [37]. Numerous studies have underscored the correlation between oxidative stress and pre-eclampsia. For instance, the fatty acid profile in women affected by pre-eclampsia may predispose them to oxidative stress, with observable increases in oxidative stress markers [38,39]. Furthermore, oxidative damage within the placenta triggers inflammation, apoptosis, and the release of cellular debris into the maternal circulation, further fueling the pathogenesis of pre-eclampsia [40]. Oxidative stress is intricately linked to the development of pre-eclampsia, with well-documented roles in causing endothelial dysfunction and instigating inflammatory responses. A comprehensive understanding of the mechanisms behind oxidative stress in pre-eclampsia is imperative for the potential development of therapeutic interventions.

Diagnostic and prognostic potential

Use of PlGF and PAPP-A as Biomarkers

PlGF and PAPP-A have emerged as promising biomarkers with potential applications in diverse pregnancy-related conditions. Extensive research suggests their diagnostic and prognostic significance in gestational diabetes mellitus (GDM), small for gestational age (SGA) infants, and specific hypertensive disorders of pregnancy. Notably, a study proposed the utility of PlGF and PAPP-A as first-trimester markers for identifying SGA infants and certain hypertensive disorders during pregnancy [3]. First-trimester screening, incorporating the measurement of these biomarkers, is under consideration for early prediction of GDM and other pregnancy complications [41]. Particularly noteworthy is the proposal to leverage PlGF and PAPP-A measurements in the first trimester, offering potential benefits in regions with limited access to regular antenatal care, primarily in developing countries [3]. However, it is crucial to acknowledge that the baseline distributions of these biomarkers may exhibit variability based on ethnicity, necessitating the consideration of specific correction factors for diverse populations [42]. While PlGF and PAPP-A are promising biomarkers for a spectrum of pregnancy-related conditions, including their potential in early prediction and management, comprehensive research is imperative. Full validation of their clinical utility, along with addressing potential variations across different populations, remains an essential avenue for future investigation.

Screening and Monitoring Strategies

Pre-eclampsia screening encompasses a range of tests and risk assessment tools designed to identify pregnant women at an elevated risk, allowing for vigilant monitoring and effective disease management. Standard screening methods include blood pressure measurement and testing for proteinuria, both deemed routine in the evaluation for pre-eclampsia. The US Preventive Services Task Force (USPSTF) has substantiated the efficacy of these methods, recognizing their accuracy in screening for pre-eclampsia. Early detection through these screening measures facilitates close surveillance and timely intervention, mitigating the potential for serious complications [43,44]. Key clinical conditions and risk factors associated with an increased likelihood of pre-eclampsia encompass a history of eclampsia or previous adverse pregnancy outcomes, maternal comorbid conditions, multifetal gestation, nulliparity, obesity, African American race, low socioeconomic status, and advanced maternal age [43]. While blood pressure measurement and proteinuria testing are firmly established as effective screening methods, the USPSTF has identified insufficient evidence regarding the effectiveness of risk prediction tools (such as clinical indicators, serum markers, or the uterine artery pulsatility index) to support varied screening strategies for pre-eclampsia [43]. Given pre-eclampsia's complex pathophysiology and clinical unpredictability, further research is imperative to refine risk-based screening approaches. This involves investigating the effectiveness of longstanding screening practices, developing novel markers, tools, or tests for early identification and disease treatment, and validating risk prediction models [45-47]. Advancements in these areas are crucial for enhancing the precision and efficacy of pre-eclampsia screening, ultimately improving outcomes for both mothers and infants.

Predictive Value for Pre-eclampsia

Numerous studies have delved into the predictive capabilities of various clinical tools and biomarkers for pre-eclampsia. One such study identified that maintaining an sFlt-1:PlGF ratio of 38 or lower proves useful in predicting the short-term absence of pre-eclampsia in women where clinical suspicion exists [48]. Another study developed and validated a first-trimester screening algorithm, amalgamating maternal characteristics, medical history, and biomarkers to predict preterm pre-eclampsia [49]. However, the intricate pathophysiology of pre-eclampsia and its diverse outcomes pose challenges for research, necessitating further investigations to comprehensively understand and define pre-eclampsia while also developing innovative screening tools and tests [45,46]. The USPSTF has underscored the adequacy of evidence supporting the accuracy of blood pressure measurement and proteinuria testing via a dipstick for pre-eclampsia screening. However, the task force has noted the lack of adequate evidence regarding the effectiveness of risk prediction tools that would endorse distinct screening strategies for pre-eclampsia [46]. The evolving landscape of pre-eclampsia research calls for ongoing studies to refine and expand our understanding of predictive tools, ultimately paving the way for enhanced screening strategies and improved clinical outcomes.

Therapeutic implications

Targeting PlGF and PAPP-A for Treatment

The therapeutic potential of PlGF and PAPP-A in pre-eclampsia is under rigorous investigation. These biomarkers have been a focal point in research for their potential applications in predicting, diagnosing, and treating pre-eclampsia. A study has demonstrated that, at a 20% false-positive rate, a substantial percentage of early-onset (71%) and preterm (58%) pre-eclampsia cases could be predicted by considering maternal characteristics, PlGF and PAPP-A [3]. However, an alternative study suggested that substituting PAPP-A for PlGF diminishes the efficacy of screening for preterm pre-eclampsia [50]. Furthermore, maternal serum levels of sFlt-1, PlGF, and PAPP-A have been correlated with late-onset pre-eclampsia and IUGR [15]. PlGF, recognized as a potent angiogenic factor, is crucial in stimulating angiogenesis in various conditions, such as heart and limb ischemia [51]. Its expression is notably downregulated in pre-eclampsia, contrasting with increased levels observed in cases of IUGR [51]. PAPP-A has been associated with the development of pre-eclampsia, with higher levels identified in pregnant women affected by pre-eclampsia compared to those without the condition [15]. Additionally, the use of aspirin (150 mg/day) has demonstrated efficacy in reducing the risk of pre-eclampsia in high-risk women [52]. Despite these findings, a comprehensive understanding of the therapeutic implications of targeting PlGF and PAPP-A in the treatment of pre-eclampsia remains an ongoing area of exploration. Further research is essential to elucidate the potential benefits and limitations, paving the way for informed and effective therapeutic interventions in the management of this complex pregnancy disorder.

Current and Potential Interventions

Potential interventions aimed at reducing the risk of pre-eclampsia encompass the use of calcium, aspirin, and anti-hypertensive agents. A comprehensive Cochrane review demonstrated that administering antiplatelet agents, primarily aspirin, was linked to a 17% reduction in the risk of pre-eclampsia [53]. Additionally, various antihypertensive agents, including beta-blockers like labetalol, have been employed in managing pre-eclampsia [53,54]. Furthermore, the utilization of aspirin at a dosage of 150 mg per day has exhibited efficacy in lowering the risk of pre-eclampsia, particularly in high-risk women [53]. While these interventions present promise in mitigating the risk of pre-eclampsia and enhancing maternal outcomes, it's crucial to acknowledge that the only definitive resolution for pre-eclampsia is the delivery or termination of pregnancy. Once a diagnosis is established, antihypertensive medications become pivotal treatments before delivery, complemented by measures for fluid control, prevention, and treatment of end-organ damage [54,55]. Nevertheless, further research is imperative to thoroughly comprehend the therapeutic implications of these interventions and refine the management strategies for pre-eclampsia. Ongoing studies in this domain aim to enhance our understanding and contribute to more effective interventions for this complex and potentially serious pregnancy complication.

Challenges and Future Directions

Pre-eclampsia continues to pose a formidable challenge in obstetrics, necessitating further research to enhance its diagnosis, management, and prevention. A key hurdle lies in the absence of a definitive diagnostic test for pre-eclampsia, making it challenging to identify at-risk women and implement timely interventions. Moreover, pre-eclampsia is a multifaceted disorder with diverse etiologies, and its pathogenesis remains incompletely understood [56]. Future research endeavors should focus on identifying novel biomarkers for pre-eclampsia, advancing the development of more precise diagnostic tests, and exploring innovative therapeutic interventions. Recent studies have explored the potential utility of microRNAs, small non-coding RNAs regulating gene expression, as biomarkers for pre-eclampsia. The ongoing investigation into the use of PlGF and PAPP-A as biomarkers for pre-eclampsia requires further research to enhance their diagnostic accuracy [49].

Another critical area of exploration is the immune system's role in pre-eclampsia development. Emerging studies propose pre-eclampsia as a potential autoimmune disorder, indicating that abnormalities in the maternal immune response may contribute to its pathogenesis. Understanding the immune system's role in pre-eclampsia may open avenues for novel therapeutic interventions. Pre-eclampsia remains a substantial challenge in obstetrics, urging ongoing research efforts to refine diagnostic approaches, enhance management strategies, and explore preventative measures. Future research directions encompass the identification of new biomarkers, the development of more accurate diagnostic tests, and the exploration of innovative therapeutic interventions [57]. Advancements in these domains are crucial for improving outcomes and addressing the complex nature of pre-eclampsia.

## Conclusions

This review has underscored the pivotal roles of PlGF and PAPP-A in the complex landscape of pre-eclampsia. The intricate interplay of these biomolecules in normal placental development and their dysregulation in pre-eclampsia have been elucidated. Importantly, altered levels of PlGF and PAPP-A emerge as promising biomarkers for early detection and diagnosis, providing valuable insights into the underlying pathophysiology. The identified pathophysiological mechanisms, including angiogenic imbalance, placental dysfunction, and inflammatory responses, shed light on the intricate molecular landscape of pre-eclampsia. Furthermore, the therapeutic potential of targeting PlGF and PAPP-A presents an avenue for future research, necessitating comprehensive investigations into effective intervention strategies. As we look ahead, the implications for future research lie in unraveling the mechanistic intricacies, validating diagnostic and prognostic potentials through longitudinal studies, refining intervention strategies, and fostering multidisciplinary collaborations. Overall, the review emphasizes the crucial importance of PlGF and PAPP-A in understanding, diagnosing, and managing pre-eclampsia, with the ultimate goal of improving maternal and fetal outcomes in pregnancies affected by this challenging condition.
